# Beneficial effects of *Cuscuta chinensis* extract on glucocorticoid-induced osteoporosis through modulation of RANKL/OPG signals

**DOI:** 10.1590/1414-431X20198754

**Published:** 2019-12-05

**Authors:** Hui Mo, Ning Zhang, Huifu Li, Fan Li, Rong Pu

**Affiliations:** 1 Department of Nuclear Medicine, Maoming People's Hospital, Maoming, Guangdong, China; 2 Clinical Laboratory of the Third People's Hospital of Dongguan City, Dongguan, Guangdong, China

**Keywords:** Dexamethasone, Oxidative status, Bone formation, Anti-osteoporosis, RunX2

## Abstract

*Cuscuta chinensis* Lam. (Convolvulaceae) is an important herbal medicine widely used to improve sexual function, treat osteoporosis, and prevent aging, and has been reported to exhibit anti-osteoporotic effects *in vitro*. However, the activity of *Cuscuta chinensis* Lam. on glucocorticoid-induced osteoporosis still remains unclear. The present study aimed to assess the protective effect and the underlying mechanism of action of *Cuscuta chinensis* extract (CCE) against glucocorticoid-induced osteoporosis *in vivo*. Sprague-Dawley rats were randomly divided into four groups as follows: control group, osteoporosis group, and 2 CCE-treated osteoporosis groups (100 mg·kg^-1^·day^-1^). Blood samples and femur bones were collected for immunohistochemistry, biochemical, mRNA expression, and western blot analysis. HPLC analysis revealed that chlorogenic acid, quercetin, and hyperin were the major constituents of CCE. The results indicated that CCE increased bone length, bone weight, and bone mineral density and suppressed dexamethasone (DEX)-induced reduction in body weight. In addition, TRAP staining indicated that CCE reduced osteoclasts in DEX-induced osteoporosis rats. Mechanistically, CCE treatment alleviated the increase of bone resorption markers and the decline of osteogenic markers, which might be partially mediated by regulation of RANKL/OPG and RunX2 pathways. These results suggest that CCE showed promising effects in the protection against glucocorticoid-induced osteoporosis through protecting osteoblasts and suppressing osteoclastogenesis.

## Introduction

Osteoporosis is a systemic metabolic disease, characterized by a decline in bone mass and deterioration of bone microstructure, which results in increased risk of bone fractures and bone fragility, and the main reason for broken bones in the elderly (1). Osteoporosis also occurs due to other diseases such as hyperthyroidism, alcoholism, oophorectomy, and kidney disease (2). Epidemiologic research has indicated synthetic drugs such as selective serotonin reuptake inhibitors, glucocorticoids, and proton pump inhibitors could lead to the induction of osteoporosis (3). Glucocorticoids are widely used in the treatment of rheumatic disease, autoimmune disease, and organ transplantation due to their immunosuppressive and anti-inflammatory effects (4). However, long-term glucocorticoid therapy is associated with an incidence of osteoporosis, which is one of themost serious side effects of glucocorticoid therapy (5). Therefore, it is important to understand the pathogenesis of glucocorticoid-induced osteoporosis and develop a novel therapeutic strategy for prevention and treatment of osteoporosis.

Although the mechanisms involved in the development of glucocorticoid-induced osteoporosis are elusive and many risk factors are related to osteoporotic fractures, oxidative stress is considered a crucial pathogenic factor of osteoporosis (6). Previous studies in animals and humans indicated that aging and its associated increase in reactive free radicals are the main reasons for decline in bone strength and mass (7). Reactive free radicals greatly impact survival and generation of osteoclasts, osteocytes, and osteoblasts (8). Previous study has indicated that dexamethasone (DEX) induces oxidative stress by promoting reactive free radicals generation or inhibiting the activities of antioxidant enzymes (9). In addition, cumulative preclinical research indicates that natural products with antioxidant activities could protect against glucocorticoid-induced osteoporosis (10).

Hence, antioxidant compounds might inhibit the development of glucocorticoid-induced osteoporosis. Alterations in the RANKL/OPG axis by glucocorticoid treatment play a vital role in the pathogenesis of glucocorticoid-evoked osteoporosis. These alterations are attributed to the ability of glucocorticoids to down-regulate osteoprotegerin (OPG) and up-regulate the receptor activator of nuclear factor kappa-Β ligand (RANKL) (10,11). However, it is mandatory to differentiate postmenopausal osteoporosis from the glucocorticoid-induced osteoporosis since they present different pathological mechanisms, especially regarding the participation of pro-inflammatory cytokines. The increased pro-inflammatory cytokines that occur postmenopause lead to bone loss by accelerating osteoclast differentiation. Glucocorticoids may be beneficial for the treatment of postmenopausal osteoporosis due to their anti-inflammatory effects. However, long-term glucocorticoid therapy is associated with the incidence of osteoporosis. Therefore, compounds that target to RANKL/OPG signal might be beneficial for glucocorticoid-induced osteoporosis treatment.

*Cuscuta chinensis* Lam. (Convolvulaceae) has been commonly used as a traditional Chinese medicine (TCM). It is also used as a functional food by adding to porridge to prevent osteoporosis, improve sexual function, and prevent aging (12). Pharmacological research has indicated that *C. chinensis* possesses hepatoprotective effects, neuroprotective effects, and antioxidant activity (12). Moreover, some studies have indicated that *C. chinensis* enhanced alkaline phosphatase (ALP) activity, BMP-2 expression, mineralization and collagen synthesis in MG-63 cells, and anti-osteoporotic effects *in vitro* (13,14). To the best of our knowledge, *C. chinensis* contains flavonoids, such as chlorogenic acid, quercetin, campherol, hyperin, among other compounds (15). Chlorogenic acid from Eucommiae improved the trabecular micro-architecture and BMD in ovariectomy-induced osteoporosis (16), and quercetin promoted osteogenic differentiation and bone marrow mesenchymal stem cells proliferation (17). However, the activities of *C. chinensis* Lam. extract (CCE) on glucocorticoid-induced osteoporosis still remain unclear. More importantly, the greatest advantage of TCM is the synergism among active ingredients. Previous study indicated QiShenYiQi pills possess a more potent cardioprotection effect than any of its compounds, due to the synergism of its active ingredients through a multi-target and multi-component mode (18).

Therefore, the present work was performed to analyze the main active compounds and investigate the protective effect of CCE against glucocorticoid-induced osteoporosis. In addition, we explored the involvement of RANKL/OPG and RunX2 signaling pathway as the underlying molecular mechanisms.

## Material and Methods

### Chemicals and drugs

Standards of chlorogenic acid (≥98%), quercetin (≥98%), hyperin (≥98%), and DEX were purchased from Sigma-Aldrich (USA). The seeds of *Cuscuta chinensis* Lam. were purchased from Tongrentang Chinese Medicine (China), and raw medicinal materials were identified by Dr. Rong Pu, according to the Pharmacopeia of the People's Republic of China. All other chemical reagents with analytical grade were purchased from Aladdin Reagent Co., Ltd (China).

### Preparation of CCE

The seeds of *Cuscuta chinensis* Lam. were ground into powder and sieved through a 20-mesh sieve. The extraction process of CCE was based on a previous publication (13). Briefly, 5 L of 95% ethanol was added to 500 g of dried powder. Then, the mixture was extracted twice at room temperature (each time was 60 min). The extract was then filtered and condensed under vacuum, and then further dried using a freeze-dryer.

### Chromatogram analysis of CCE by HPLC-UV

The chromatogram analysis was performed on an Agilent 1260 system equipped with Agilent ZORBAX SB C18 column (50×4.6 mm, 5 μm) and quaternary pumps. The column temperature was 35°C and flow was set at 0.6 mL/min. The mobile phase consisted of 0.1% formic acid (A) and acetonitrile (B) that were applied as follows: 95 (A) to 70% (A) for 15 min, and 5 (B) to 30% (B) held for 10 min.

### Animals

The animal experiments were approved by the Animal Care and Use Committee and performed in accordance with local institutional ethics. Sprague-Dawley rats (350±20 g, five months old) were purchased from Beijing HuaFuKang Bioscience Co., Ltd. (China) and were housed under standard pathogen-free conditions (air temperature 20–22°C, humidity 45–55%, and 12-h light/dark cycle). All rats were fed with a basic diet for 7 days to adapt to the laboratory condition.

### Experimental design

#### Induction of osteoporosis and treatments

Sprague-Dawley rats were randomly divided into four groups (n=8) as follows: control group (CON), osteoporosis group (DEX), and 2 groups of rats with osteoporosis receiving 100 mg/kg CCE (DEX+CCE); the dose of CCE (100 mg/kg) was based on previous research (19) and our preliminary experiments. Rats in all groups except control group were injected with 7 mg/kg dexamethasone disodium phosphate intramuscularly once a week for five weeks (20). Rats in the DEX+CCE (i) group received three weeks of 100 mg/kg CCE intragastrically once a day starting the second week of osteoporosis induction; rats in the DEX+CCE (ii) group received three weeks of 100 mg/kg CCE intragastrically once a day starting one week before osteoporosis induction. The CON group and DEX group received the same amount of physiological saline. At the end of the fifth week, blood samples were obtained from the eyeballs of the animals. Afterwards, all rats were euthanized. The soft tissues around the femur were removed carefully. The left femur bones were evaluated for length and wet weight, while the right femur bones were stored in –20°C for further measurement.

### Immunohistochemical staining for tartrate-resistant acid phosphatase (TRAP)

Femoral bone tissue was decalcified using decalcifying solution and dehydrated using ethanol solutions for 18 h. Then, the bone tissue was embedded in paraffin and cut into 5-µm thick sections. Histochemical staining was performed using leukocyte acid phosphatase kit (Nanjing Jiancheng Bioengineering Institute, China) according to the manufacturer's protocol. The sections were observed under a light microscope (100× magnification).

### Bone mineral density (BMD)

The BMD of the femur was assayed by dual energy x-ray absorptiometry (GE Healthcare, USA) and is reported as g/cm^2^.

### Measurement of bone tissue oxidative stress

The activities of superoxide dismutase (SOD), glutathione peroxidase (GSH-Px), and catalase (CAT), and the content of malondialdehyde (MDA) were measured using a commercial kit (Nanjing Jiancheng Bioengineering Institute) according to the manufacturer's protocol.

### Analysis of serum insulin-like growth factor (IGF) and transforming growth factor (TGF)-β

The serum levels of IGF and TGF-β were assayed with the rat ELISA kit (Nanjing Jiancheng Bioengineering Institute) according to the manufacturer's instructions.

The activities of TRAP and alkaline phosphatase (ALP) were measured using a commercial kit (Nanjing Jiancheng Bioengineering Institute) according to the manufacturer's protocol. Briefly, serum samples were incubated with the test solutions at 37°C for 1 h. Finally, absorbance was assayed using a microplate reader (BioTek Epoch, USA) at 530 nm (TRAP) or 520 nm (ALP). Osteocalcin (OCN) and C-terminal telopeptide of type I collagen (CTX) serum levels were determined using a commercial kit (USCN Life Science, China) according to the manufacturer's protocol. Briefly, serum samples were mixed with the antibody solutions (OCN and CTX) at 37°C for 1 h. After streptavidin-HRP was added, the solution was incubated at 37°C for 30 min. Absorbance was assayed using a microplate reader at 450 nm.

### Toxicity study

Serum aspartate aminotransferase (AST), alanine aminotransferase (ALT), serum creatinine (CRE), and serum urea nitrogen (BUN) were measured by a commercial assay kit (Nanjing Jiancheng Bioengineering Institute) according to manufacturer's instructions.

### Quantitative real-time polymerase chain reaction (qRT-PCR)

The mRNA expression of OPG, RANKL, runt-related transcription factor 2 (RunX2), and OCN in tissue from the right femur was assayed by RT-qPCR. Briefly, total RNA was isolated from frozen femur tissue using Trizol reagent (Takara, China) according to the manufacturer's protocol. The cDNA was synthesized from total RNA using the First Strand cDNA Synthesis kit (Thermo Fisher Scientific, USA). RT-PCR was performed with a SYBR Green qPCR Master Mix kit (Thermo Fisher Scientific). The qPCR was performed in duplicate, the condition of RT-PCR amplification reaction was as follows: 40 cycles of 95°C for 10 s, 60°C for 30 s, and 72°C for 15 s with the primer sequences (Table 1); GAPDH acted as an internal control. The relative mRNA expression was calculated by the 2^−ΔΔCT^ method.

**Table 1.** Primer sequences for quantitative real-time RNA.

Genes	Forward primer	Reverse primer
OPG	5′-TACAGCATCACTACGTAGGAC-3′	5′-ACGTCATGCGATCACATATCG-3′
RANKL	5′-GACAGGCACGGACTCGTA-3′	5′-CGCTCATGCTAGTCGTCTA-3′
Runx2	5′-AGCGCTTCTCAGGAGTTCCA-3′	5′-GCCGGGCCACATCGA-3′
OCN	5′-CTGGCTGCGCTCTGTCTCT-3′	5′-TGCTTGGACATGAAGGCTTTG-3′
GAPDH	5′-CAACTTTGGCATTGTGGAAGG-3′	5′-ACACATTGGGGGTAGGAACAC-3′

### Western blotting

The right femur tissues were homogenized in RIPA buffer using a homogenizer and the homogenate was centrifuged at 12,000 *g* for 15 min at 4°C. Total proteins of the supernatant were measured using the Bicinchoninic acid Protein Assay Kit (Thermo Fisher Scientific). Protein samples were separated by 15% SDS-polyacrylamide gels, and then transferred onto a PVDF membrane (Roche, USA). After blocking with 5% skim milk for 1 h at room temperature, the membrane was hybridized with the following primary antibodies overnight at 4°C: anti-OPG (1:1,000; Abcam, USA), anti-RANKL (1:1,000; Abcam), anti-RunX2 (1:1,000; Abcam), anti-OCN (1:1,000; Abcam), and GAPDH (1:1,000; Abcam). The membrane was washed with TBST and subsequently incubated with horseradish peroxidase-labeled secondary antibody for 45 min at room temperature. Visualization was performed with chemiluminescence detection reagent (ECL, USA). The relative protein levels were analyzed with ImageJ image analysis software (National Institutes of Health, USA) and normalized to GAPDH.

### Effects of CCE on anti-inflammatory effect of DEX

Rats were randomly divided into five groups (n=8) and pre-treated with either blank solution or corresponding drugs (CCE 100 mg/kg or DEX 40 mg/kg or CCE 100 mg/kg + DEX 40 mg/kg of body weight) dermally once daily for four days. Paw edema was evoked by subcutaneous injection of 1% carrageenan solution (0.1mL) one hour after the last drug treatment. After 30 min, the rats were treated with corresponding drugs again. Paw edema was measured 6 h after the carrageenan induction.

Tissue samples were obtained from paw edema and homogenized after adding normal saline at a ratio of 1:9 (w/v). The homogenate was centrifuged at 4000 *g* for 10 min at 3°C and the supernatant was collected and stored at -80°C until further analysis. The pro-inflammatory cytokines interleukin (IL)-1β, IL-6, and tumor necrosis factor alpha (TNF-α) were measured using a commercial kit (Nanjing Jiancheng Bioengineering Institute) according to manufacturer's instructions.

### Statistical analysis

Data are reported as means±SD. Differences between groups were compared using one-way ANOVA followed by Tukey's multiple comparison test for *post hoc* analysis using GraphPad Prism software (USA). P<0.05 was considered statistically significant.

## Results

### HPLC-UV profiles of CCE extract

**Figure 1.**
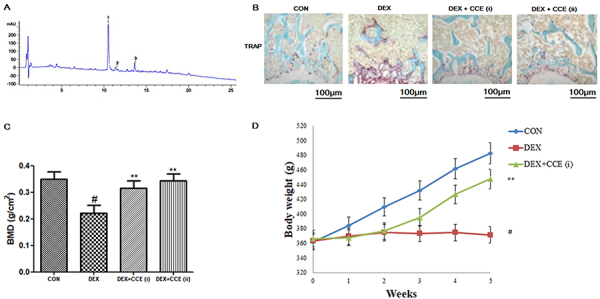
**A**, Chromatograms of flavonoids occurring in hydroalcoholic extract of *Cuscuta chinensis*. (1: chlorogenic acid, 2: quercetin, and 3: hyperin). **B**, Representative immunohistochemical staining images for TRAP in femoral bone tissue sections (100× magnification, bar: 100 μm). Effects of *Cuscuta chinensis* extract (CCE) on the changes in bone mineral density (BMD) (**C**) and body weight (**D**) in dexamethasone (DEX)-induced osteoporosis in rats. Data are reported as means±SD (n=8/group). ^#^P<0.05 *vs* the CON group, **P<0.05 *vs* the DEX group (ANOVA).

As shown in Figure 1A, HPLC-UV analysis indicated the major bioactive constituents in CCE extract. The three observed flavonoids were assigned as chlorogenic acid, quercetin, and hyperin by comparing the retention times with the reference in HPLC chromatograms.

### Effect of CCE on osteoclast activity

As shown in Figure 1B, TRAP staining indicated larger osteoclasts in DEX-induced osteoporosis rats compared to control rats. However, the marker was normalized by treatment with CCE. The result indicated that CCE suppressed osteoclastic bone resorption in DEX-induced osteoporosis in rats.

### Effects of CCE on the changes in BMD and body weight

DEX-induced osteoporosis was successfully established by intramuscular injection of DEX. As shown in Figure 1C, consistent with previous research, the BMD index was reduced in the DEX group (21), which was partially alleviated by treatment with CCE. As shown in Figure 1D, in line with the reduction of the BMD index, body weight was decreased in the DEX group, however, with the treatment of CCE, body weight was significantly increased compared to the DEX group (P<0.05). Hence, our preliminary data implied that treatment with CCE did not decrease body weight, and showed no observable toxicity.

### Effects of CCE on DEX-induced bone physical change

**Figure 2.**
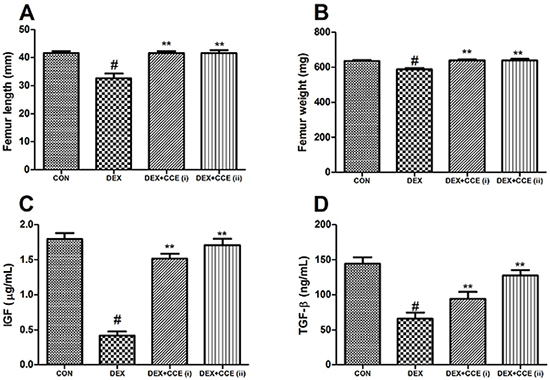
Effects of *Cuscuta chinensis* extract (CCE) on femur length (**A**), femur weight (**B**), serum insulin-like growth factor (IGF) (**C**), and serum transforming growth factor (TGF-β) (**D**) in dexamethasone (DEX)-induced rats. Data are reported as means±SD (n=8/group). ^#^P<0.05 *vs* the CON group, **P<0.05 *vs* the DEX group (ANOVA).

As shown in Figure 2A and B, administration of DEX alone resulted in a decline in femur length and weight compared to the control group (P<0.05). However, treatment with CCE ameliorated the DEX-induced reduction of femur length and weight compared to the DEX group (P<0.05).

### Effects of CCE on bone metabolism

**Figure 3.**
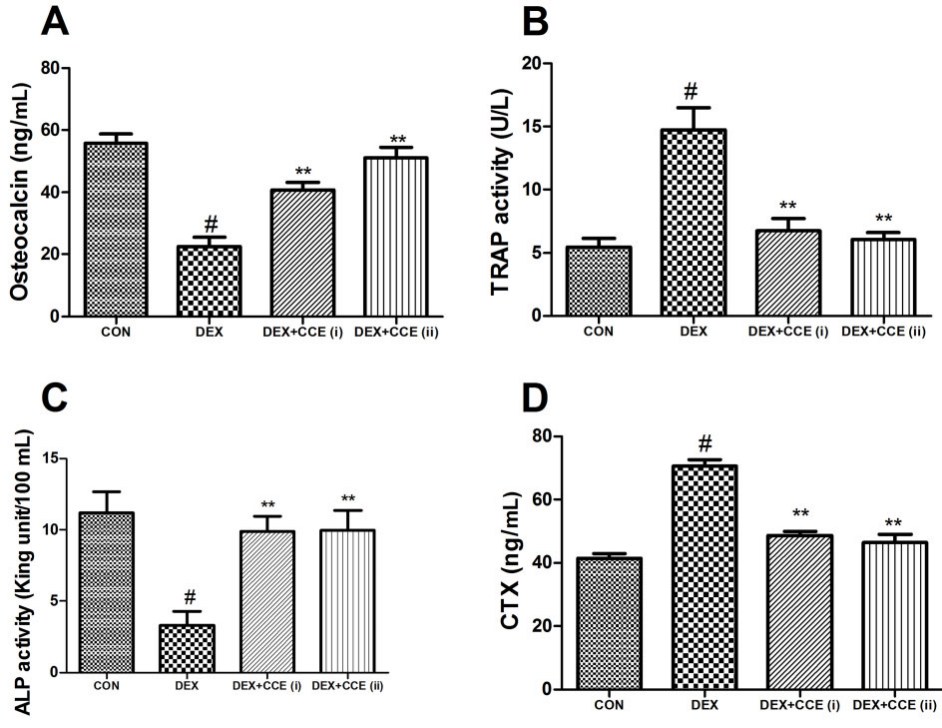
Effects of *Cuscuta chinensis* extract (CCE) on bone turnover markers in dexamethasone (DEX)-induced rats. **A**, Serum levels of osteocalcin. **B**, Immunohistochemical staining for tartrate-resistant acid phosphatase (TRAP) activity in serum. **C**, Alkaline phosphatase (ALP) activity in serum. **D**, Serum levels of C-terminal telopeptide of type I collagen (CTX). Data are reported as means±SD (n=8/group). ^#^P<0.05 *vs* the CON group, **P<0.05 *vs* the DEX group (ANOVA).

As shown in Figure 2C and D and Figure 3, the levels of IGF, TGF-β, OCN, and activity of ALP in serum were decreased and the CTX content and activity of TRAP in the serum were increased in the DEX group compared to the control group (P<0.05). However, with the treatment of CCE, these changes were reversed.

### Effects of CCE on oxidative stress

**Figure 4.**
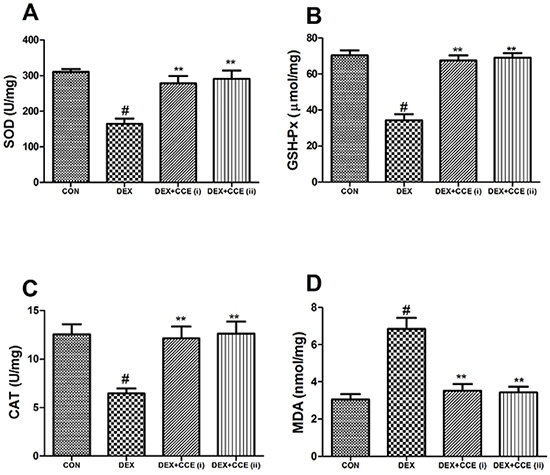
Effects of *Cuscuta chinensis* extract (CCE) on superoxide dismutase (SOD) activity (**A**), glutathione peroxidase (GSH-Px) activity (**B**), catalase (CAT) activity (**C**), and malondialdehyde (MDA) level (**D**) in femur tissue of dexamethasone (DEX)-induced osteoporosis rats. Data are reported as means±SD (n=8/group). ^#^P<0.05 *vs* the CON group, **P<0.05 *vs* the DEX group (ANOVA).

As shown in Figure 4, in the DEX group, the activity of SOD, GSH-Px, and CAT was lower and the content of MDA in the femur tissue was higher compared to the control group (P<0.05). However, treatment with CCE improved the activity of SOD, GSH-Px, and CAT and decreased the content of MDA in the CCE group.

### Effects of CCE on DEX-induced changes in mRNA expression and protein levels

**Figure 5.**
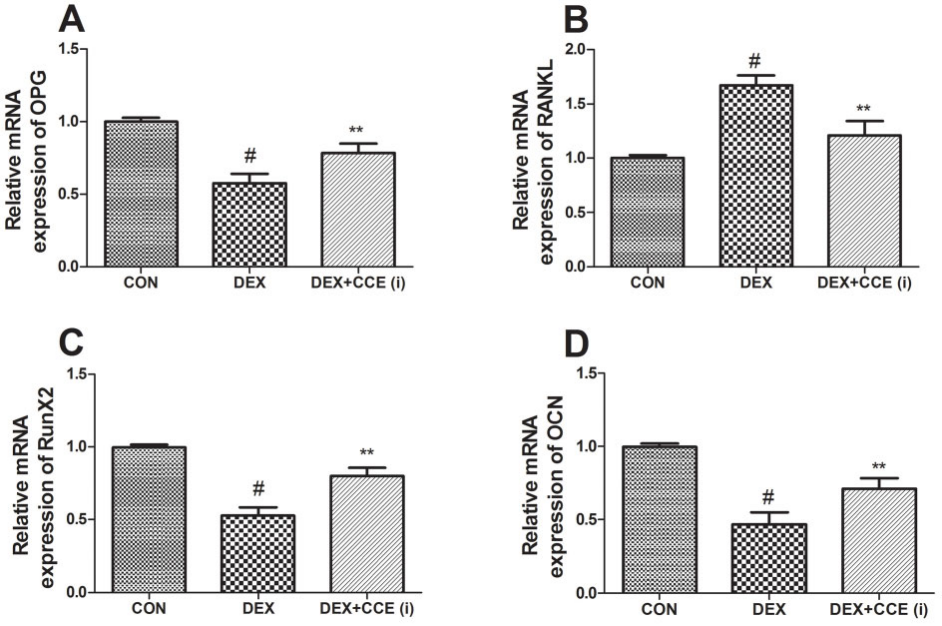
Effects of *Cuscuta chinensis* extract (CCE) on mRNA expression of osteoprotegerin (OPG) (**A**), receptor activator of nuclear factor kappa-Β ligand (RANKL) (**B**), RunX2 (**C**), and osteocalcin (OCN) (**D**) in dexamethasone (DEX-induced osteoporosis rats. Data are reported as means±SD (n=8/group). ^#^P<0.05 *vs* the CON group, **P<0.05 *vs* the DEX group (ANOVA).

**Figure 6.**
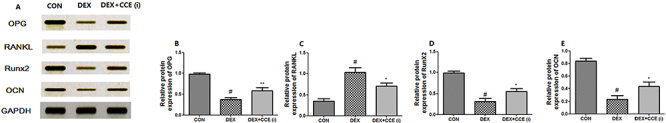
Effects of *Cuscuta chinensis* extract (CCE) on protein expression (**A**) of OPG (**B**), receptor activator of nuclear factor kappa-Β ligand (RANKL) (**C**), RunX2 (**D**), and osteocalcin (OCN) (**E**) in dexamethasone (DEX)-induced osteoporosis rats. Data are reported as means±SD (n=8/group). ^#^P<0.05 *vs* the CON group, **P<0.01, *P<0.05 *vs* the DEX group (ANOVA).

As shown in Figures 5 and 6, mRNA expression and protein levels of OPG, RunX2, and OCN were decreased and the mRNA expression and protein level of RANKL was increased in the DEX group compared to the control group (P<0.05). However, CCE treatment markedly up-regulated OPG, RunX2, and OCN and down-regulated RANKL mRNA expression and protein levels.

### Toxicological effect of CCE

**Figure 7.**
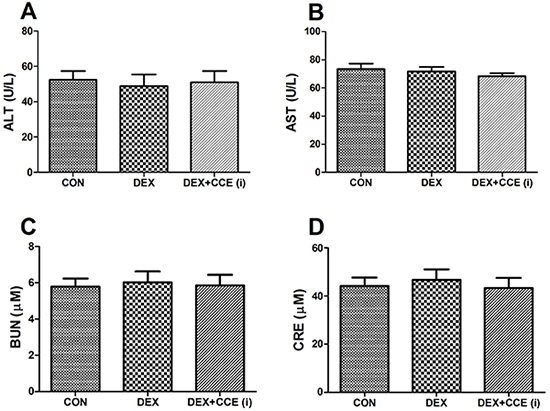
Effects of *Cuscuta chinensis* extract (CCE) on the serum levels of alanine aminotransferase (ALT) (**A**), aminotransferase (AST) (**B**), serum urea nitrogen (BUN) (**C**), and creatinine (CRE) (**D**) in dexamethasone (DEX)-induced osteoporosis rats. The data are reported as means±SD (n=8/group) (P>0.05, ANOVA).

As shown in Figure 7, CCE treatment had no apparent effect on hepatic function and renal function indicators compared with the CON group (P>0.05). These results indicated that the administration of CCE showed very little toxicological effects in DEX-induced osteoporosis rats.

### Effects of CCE on anti-inflammatory effect of DEX

**Figure 8.**
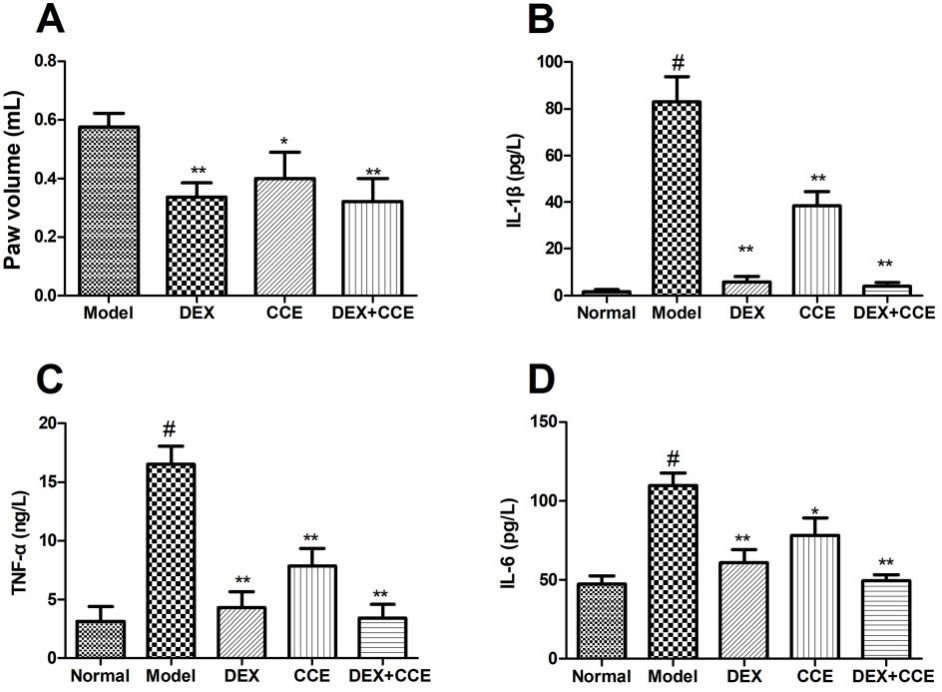
Effects of *Cuscuta chinensis* extract (CCE) on anti-inflammatory effect of dexamethasone (DEX). **A**, paw volume; **B**, interleukin 1-β (IL-β); **C**, tumor necrosis factor alpha (TNF-α); **D**, IL-6. Data are reported as means±SD (n=8/group). ^#^P<0.05 *vs* the normal group, **P<0.01, *P<0.05 *vs* the model group (ANOVA).

As shown in Figure 8, topical application of DEX+CCE suppressed edema formation and decreased IL-1β, TNF-α, and IL-6 levels. The inhibition effects of DEX+CCE were comparable to those of DEX (P>0.05). Therefore, CCE treatment had no negative effect on the effects of DEX in acute models of inflammation.

## Discussion

Glucocorticoids have been extensively used in the treatment of auto-immune and rheumatic diseases, and acute medical conditions, such as traumatic spinal cord damage (22). However, glucocorticoid overuse often causes side effects on the vascular system, resulting in decreased blood supply in the femoral head (23). In addition, glucocorticoids have been reported to suppress the osteogenic differentiation of mesenchymal stem cells. The inhibitive effect of glucocorticoids may be related to the induction of down-regulation of RunX2, which is deemed to be important in the development of osteoblasts (24). Furthermore, glucocorticoid-evoked oxidative stress accelerates apoptosis of osteoblasts, leading to the suppression of osteoblastic proliferation (25). Therefore, natural products have been recommended to suppress bone resorption and osteoclast differentiation due to their antioxidant effect (10). DEX, a common glucocorticoid used in clinics, shows deleterious effects on bone minerals, turnover, and structure. Therefore, in the present study, rats received injection of DEX intramuscularly once a week for five weeks to establish the rat model of osteoporosis *in vivo*.

Osteoporosis is characterized by a decrease in bone strength and bone weight, which result in an increased risk of fracture (26). BMD loss and body weight reduction often occurs after glucocorticoid treatment. BMD and bone quality are still used as the main markers of osteoporosis (27). In accordance with previous studies (27,28), our results indicated that DEX decreased BMD and suppressed body weight gain. CCE treatment alleviated DEX-induced declines in BMD and body weight gain, indicating the osteoporosis prevention effect of CCE.

TGF-β and IGF cooperate to regulate osteoblast function. TGF-β could promote the synthesis of type I collagen and improve bone regeneration. IGF regulates the stability, location, and transcriptional activity of β-catenin (29). In the present study, CCE increased the TGF-β and IGF levels in DEX-induced osteoporosis rats.

Bone homeostasis is a balance between osteoclast-regulated bone resorption and osteoblast-regulated bone formation, while disruption of this equilibrium is considered the main reason for the pathogenesis of osteoporosis (30). In addition, glucocorticoids lead to osteoporosis via various pathways, including both bone resorption and bone formation (31). In the current study, the bone formation indicators OCN and ALP were measured. OCN is a late index of osteoblast activity and is considered the richest non-collagenous protein in bone (32). ALP is an important index in the early stage of osteogenic differentiation, which participates in skeletal calcification by increasing the local content of inorganic phosphate (33). In accordance with previous reports (10,27), we observed that CCE prevented the suppression of the bone formation markers induced by DEX. TRAP is evoked in differentiation of osteoclasts and a highly expressed iron-binding protein in osteoclasts (34). CTX is another important bone resorption marker (27). In accordance with previous reports (10,27), we observed that DEX decreased the activity of TRAP and content of CTX in serum. Treatment with CCE mitigated DEX-induced increase of bone resorption markers.

Previous research showed that oxidative stress is another reason contributing to osteoporosis (35). Cumulative evidence indicated that increased ROS activity and antioxidant decrease often occurred during osteoporosis (36). In accordance with previous report (10), we observed increased oxidative stress with a rise in MDA levels in DEX-induced osteoporosis. Along with these alterations, we also found a decrease of antioxidant enzyme activities. Concurrent treatment with CCE improved oxidative stress in femur tissue. These findings indicate the antioxidant effects of CCE (37).

OPG and RANKL are two important cytokines expressed by osteoblasts that play a vital role in bone metabolism (21). RANKL facilitates bone resorption by combining with its receptor on osteoclasts. OPG combines with RANKL preventing it to combine with RANK, consequently suppressing bone resorption (38). In accordance with previous studies (10,21), we observed down-regulated OPG expression and up-regulated RANKL expression evoked by DEX. Our finding indicated the role of CCE in the suppression of DEX-evoked bone resorption.

RunX2 is a vital modulator of bone formation and osteoblast differentiation. Decline in expression of major transcription factor further resulted in reduction in OCN expression, which is an important extracellular matrix protein for bone (39). Glucocorticoid-evoked down-regulation of RunX2 and OCN expression has also been reported in previous studies (27,40). Similarly, our results showed that RunX2 and OCN expression levels were lower in femur tissue from DEX-induced osteoporosis rats. Concurrent treatment with CCE replenished RunX2 and OCN expression in femur tissue. These results may show the potential mechanism of CCE in the prevention of osteoporosis.

HPLC analysis of CCE revealed the presence of chlorogenic acid, quercetin, and hyperin. Previous research has indicated that flavonoid extracts from *Cuscuta chinensis* are responsible for the anti-osteoporosis effect *in vitro*, with which our findings are in accordance (13). In addition, chlorogenic acid prevented osteoporosis through the Shp2/PI3K/Akt pathway (16). Quercetin improved bone structure and function in post-menopausal osteoporosis rats by suppressing the TNF-α-induced NF-κB/β-catenin pathway (17).

In conclusion, our findings indicated that CCE alleviated DEX-evoked osteoporosis by suppressing bone resorption and improving bone formation. The potent antioxidant effect of CCE alleviated the progress of osteoporosis partly through modulation of RANKL/OPG and RunX2 signals. Therefore, CCE may be a promising candidate drug to prevent and treat DEX-evoked osteoporosis. Further investigations addressing its application in bone diseases are likely to provide more insight into its anti-osteoporosis effects.
